# Identification of Androgen Receptor Splice Variants in the Pten Deficient Murine Prostate Cancer Model

**DOI:** 10.1371/journal.pone.0131232

**Published:** 2015-07-21

**Authors:** Mengmeng Liang, Helty Adisetiyo, Xiuqing Liu, Ren Liu, Parkash Gill, Pradip Roy-Burman, Jeremy O. Jones, David J. Mulholland

**Affiliations:** 1 Icahn School of Medicine at Mount Sinai, New York, New York, United States of America; 2 Children’s Hospital of Los Angeles, Los Angeles, California, United States of America; 3 St. Luke's Hospital, Internal medicine resident, Chesterfield, Missouri, United States of America; 4 Department of Medicine, University of Southern California Keck School of Medicine, Los Angeles, California, United States of America; 5 Department of Biochemistry and Molecular Biology, University of Southern California, Los Angeles, California, United States of America; 6 Beckman Research Institute, City of Hope, Duarte, California, United States of America; Innsbruck Medical University, AUSTRIA

## Abstract

Androgen receptor (AR) variants are associated with resistance to anti androgen therapy both in human prostate cancer cell lines and clinical samples. These observations support the hypothesis that AR isoform accumulation is a consequence of selective therapeutic pressure on the full length AR. The Pten deficient prostate cancer model proceeds with well-defined kinetics including progression to castration resistant prostate cancer (CRPC). While surgical castration and enzalutamide treatments yield an initial therapeutic response, *Pten^-/-^*epithelia continue to proliferate yielding locally invasive primary tumor pathology. That most epithelium remains AR positive, but ligand independent, suggests the presence of oncogenic AR variants. To address this hypothesis, we have used a panel of recently described *Pten^-/-^* tumor cell lines derived from both from hormone intact (E4, E8) and castrated Pten mutants (cE1, cE2) followed by RACE PCR to identify and characterize three novel truncated, amino terminus containing AR variants (mAR-Va, b, c). Variants appear not only conserved throughout progression but are correlated with nearly complete loss of full length AR (AR-FL) at castrate androgen levels. The overexpression of variants leads to enhanced transcriptional activity of AR while knock down studies show reduced transcriptional output. Collectively, the identification of truncated AR variants in the conditional PTEN deletion model supports a role for maintaining the CRPC phenotype and provides further therapeutic applications of this preclinical model.

## Introduction

Prostate cancer cells have been hypothesized to progress to castration resistance through a multitude of androgen receptor (AR) modifications including amplification, gene rearrangements or mutations, altered expression of transcriptional co-regulators and *de novo* steroidogenesis [1–6]. More recently, studies with human prostate cancer cell lines and tissues have identified the presence of AR splice variants (AR-Vs) which can be up-regulated in response to castration and promote resistance to androgen receptor targeted therapy [7–9] [10] [11]. The androgen receptor belongs to the steroid receptor transcription factor family and is composed of an N-terminal transcriptional activation domain (NTD, exon1), DNA-binding domain (DBD, exon 2 and 3), a hinge region (exon 4), and the C-terminal ligand binding domain (LBD, exon 4–8) [12,13]. To date up to 15 different AR-Vs lacking different portions of LBD have been reported in human prostate cancer cell lines CWR-R1, 22rv1, VCaP and LuCaP xenografts [14–21]. Despite this, AR isoforms remain vastly understudied in preclinical mouse models of prostate cancer tissues being limited to two AR-Vs as reported using the Myc-CaP cell line [15]. To date, no AR variants have been reported from the primary organ of a preclinical mouse model of prostate cancer.

The *Pten* null mouse model of prostate cancer is poorly responsive to androgen ablation therapy including surgical castration and androgen receptor targeted therapy [22] [23] [24]. While heightened PI3K/Akt signaling has been shown to provide significant survival cues under castrated conditions, most epithelia remain androgen receptor positive. This observation raises the possibility that ligand independent forms of the androgen receptor may exist facilitating survival and continued progression. We have taken a multi-pronged approach to address this hypothesis including the characterization and application of several novel, Pten deficient, murine prostate cancer cell lines [25] [26] and corresponding primary tumor model.

We have identified three novel variants of the androgen receptor (termed mAR-Va, b, c). These AR variants all contain the AR-NTD with only one containing a partial sequence overlap with the AR-LBD. The relative expression of these variants not only increases in response to castration but in response to AR targeted therapy including casodex and enzalutamide. Remarkably, after progression at long term of castrate androgen levels, we observed a significant reduction in full length AR but maintenance of the identified isoforms. Functional studies revealed that overexpression or knock down of AR-Va and AR-Vc could regulate AR transcriptional output.

Collectively, our data support the presence of new AR variants that may serve as important contributors during progression to CRPC. Variant identification also significantly enhances the utility of the PTEN null prostate cancer model both in terms of understanding the function of AR and as a system for assessing therapies targeting resistant forms of the androgen receptor.

## Results

### Identification of novel AR variants in cell lines derived from the Pten null prostate cancer model

**Fig 1:**
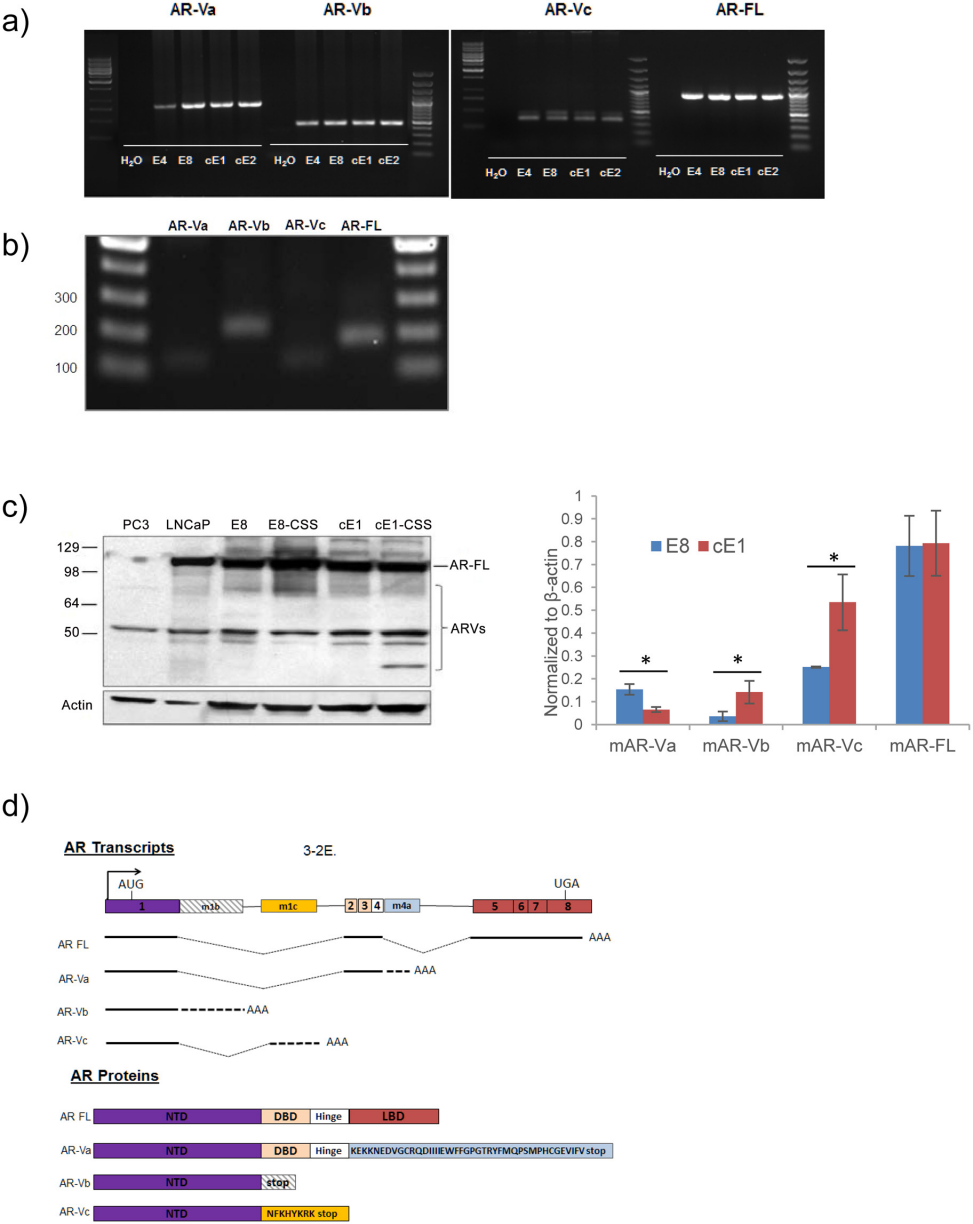
Identification of AR splice variants in cell lines derived from the Pten null prostate cancer model. **a)** Identification of mAR variants (a, b, c) in derived cell lines visualized using conventional PCR. **b)** Real time PCR evaluation of AR variants amplified from E8 cell cDNA visualized by gel electrophoresis. **c, left)** Detection of AR isoforms in human and mouse prostate cancer cell lines and **c, right)** densitometry analysis of variant and AR-FL protein expression normalized to β-actin (*, p<0.05). **d)** Schematic structure of mAR variants (a, b, c) and AR full length (AR-FL). Solid-colored boxes represent the common exons of AR-FL and hatched cassettes indicate the cryptic exons. Bold straight lines refer to the transcribed sequences in their mRNAs. The predicted amino acid sequence translated by m4a and m1c is listed (lower diagrams).

As a part of our studies of androgen receptor (AR) function and mechanisms of CRPC progression, we recently characterized four new murine prostate cancer cell lines derived from either hormone intact (E4, E8) or castrated (cE1, cE2) *Pten* null mutant mice. Cell lines were characterized as being *Pten* ^*-/-*^, having activated PI3K/Akt signaling and features of the primary tumor model [25] [26]. To assay for the presence of androgen receptor (AR) variants, we applied 3’ RACE on cDNAs generated from the E8 and cE1 lines using forward primers anchored within the exon1 of mouse AR (S1 Table). Using this unbiased approach, we amplified AR mRNAs with poly(A) tails including those AR-Vs with potential novel 3’ sequences. Following with nested PCR, we observed several unique sequence products (Fig 1a, S1 Fig). As anticipated, the longest (~2 kb) band was the full length AR (AR-FL), containing sequences upstream of the poly(A) region that matched with the 3’UTR of mouse AR (mAR) mRNA. Cloning and sequence analyses of the ~850 bp PCR products indicated the existence of several unique mouse AR-Vs (mAR-Vs) in E8 and cE1 cells. We focused on three unique transcripts. First, a short AR isoform found in the E8 line, retaining exons 1–4 from mAR mRNA, followed by a unique 175-bp sequence found to align to the AR intron region adjacent to exon 4 which we termed exon 4a (m4a) or variant AR-Va (Fig 1a and 1b). The molecular structure AR-Va is analogous to the 5’–retaining exons 1–4 but differing in the compositions of 3’-sequences of previously described mouse mAR-V4 [15] and human ARV^567es^ [27]. Next, two novel AR-Vs with potentially unique alternative splicing sites were detected in cE1 cells. When mapping their sequences to the mouse genome, we found that 442-bp and 495-bp sequences matched to intron 1 and spliced exon 1, which we referred to m1b and m1c variants. To avoid potential confusion with the nomenclature of ARVs reported in human prostate cancer models, we named these three novel AR variants as mouse AR-Va, b, c (mAR-Va, b, c), of which mAR-Va was first identified in E8, with mAR-Vb and mAR-Vc in cE1 (S1 Fig). Using the above cell lines we determined the relative expression levels of each variant. Interestingly, mAR-Va expression was highest in the E4 and E8 lines while mAR-Vb and mAR-Vc showed higher expression in the cE1 and cE2 lines. All variants were significantly lower in expression than the AR-FL (S2 Fig).

Using ApE plasmid editor software we were able to deduce the molecular weights of mAR-Va as ~80 KDa and mAR-Vb, Vc to be ~54 and 56 kDa corresponding to our identified transcripts (Fig 1d, S2 Table, S3 Table). To ascertain whether AR-Vs could be detected at the protein level we used an antibody targeting the animo terminus of the AR (Santa Cruz, N-20) and examined lysates from the E8, cE1 and castrated androgen derivatives (E8-CSS, cE1-CSS). Assessed alongside the human lines, LNCaP and PC3, we detected the AR-FL (~110 kDa) and three major bands residing at ~75 kD, 85kD and lower species at ~50kD and ~56 kD (Fig 1c, S2 Table).

### Androgen ablation increases the relative expression of AR variants

**Fig 2:**
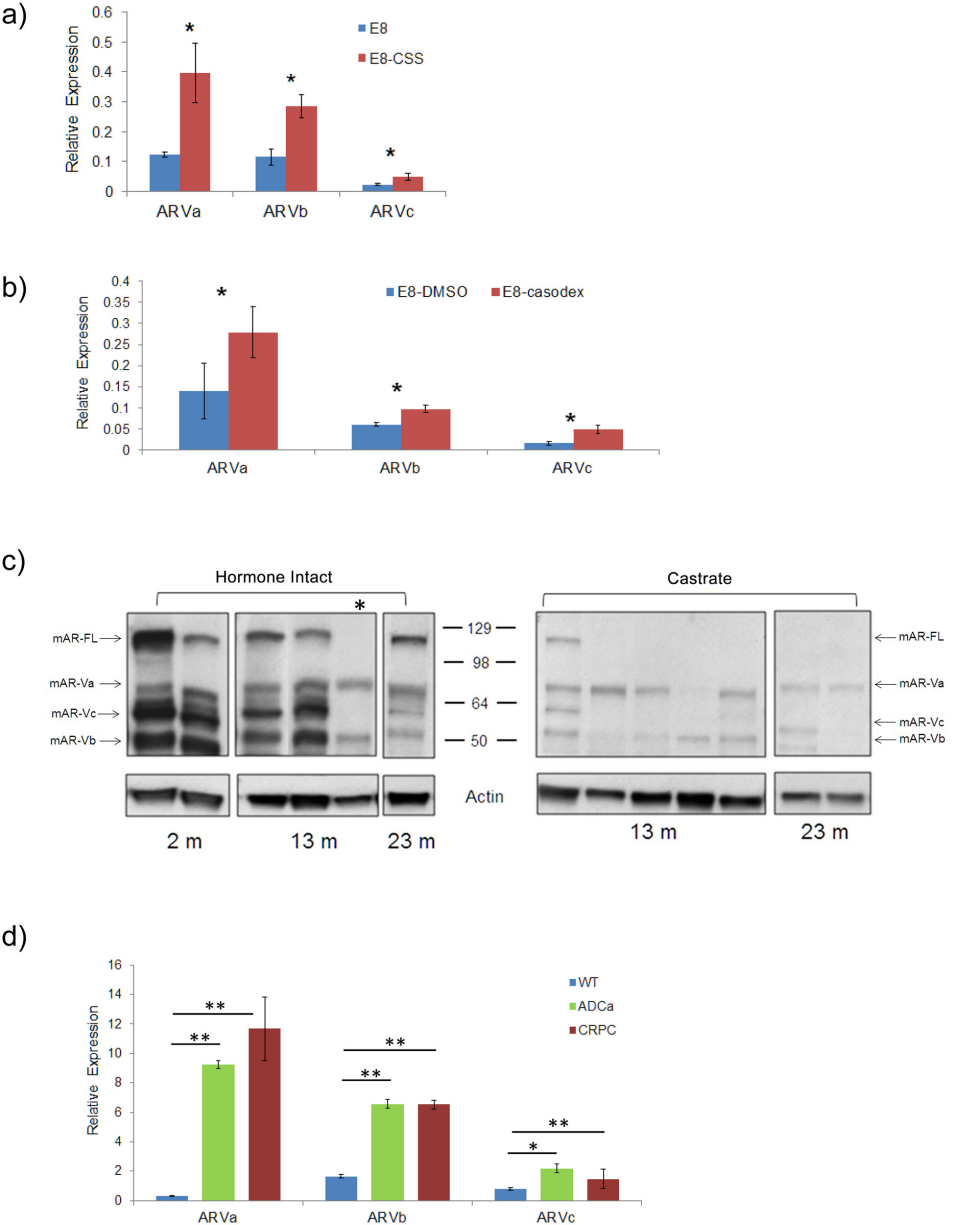
Androgen ablation increases the relative expression of androgen receptor isoforms. **a)** Culture of murine prostate cancer (E8) cells lines with anti androgen treatment including charcoal stripped serum (CSS) (3 days) or **b)** casodex (10 μM, 3 days) results in significantly enhanced expression of AR isoforms normalized to AR full length. **c)** Surgical castration of Pten mutants (*C* ^*+*^ *;Pten* ^*L/L*^, n = 7) results in a significant increase in the relative expression of AR isoforms as detected by western blotting. **d)** AR variant transcript expression from *Wt*, hormone intact and castrated mutant mice normalized to AR-FL levels (*, p<0.05; **, p<0.01).

To further support a role for the AR variants during CRPC, we considered the impact of anti-androgen treatment. To do this, we challenged the E8 and cE2 cells lines with either castrate androgen culture conditions (charcoal-stripped serum, CSS) or casodex (10 μM) for 3 days. Strikingly, in CSS culture conditions we observed increased transcript expression in all three variants with the greatest fold increase observed in mAR-Va in E8 cells (mAR-Va, 3.5 fold, mAR-Vb, 2.5 fold, mAR-Vc 2.2 fold, p < 0.05) when normalized to full length AR (Fig 2a). Using casodex, we also observed a statistically significant enhancement of all AR variants using the E8 and cE2 lines (Fig 2b).

We then considered whether AR isoforms existed *in vivo* using hormone intact Pten-null tumors (*C* ^*+*^ *;Pten* ^*L/L*^; n = 6) and whether progression at castrate androgen levels could alter their expression. Using resected lobes of the prostate (ventral, dorsal lateral, anterior) we assessed expression of ARs in hormone intact mutants at 2, 13 and 23 months and detected the presence of AR-FL (~110 kD) and three major variants of reduced size of approximately 45–50, 64 and 75 kD (Fig 2c). Of the 6 mutants analyzed, we observed only one in the 13 month cohort with reduced AR-FL expression (*, star). Strikingly, in Pten mutants that were surgically castrated (*C* ^*+*^ *;Pten* ^*L/L*^; n = 7) we observed 6/7 to have significant loss of AR-FL but maintained expression of AR isoforms (Fig 2c, right). Quantitative RNA (q-PCR) measurement of mAR-Va, b, and c confirmed the elevation of isoforms in hormone intact and CRPC Pten deficient cancers with respect to *Wt* prostates and normalized to AR-FL (Fig 2d) (**, p<0.01). To further associate the presence of truncated AR-Vs with castration resistant progression, we treated Pten hormone intact mutants (*C* ^*+*^ *;Pten* ^*L/L*^, n = 6) with enzalutamide (10 mg/kg, *via* mouse chow *ad libitum*) for 10 weeks, followed by immunohistochemistry using antibodies against either the AR amino terminus (AR, N-20) or AR c-terminus (AR, C-19). Consistent with biochemical analysis, we observed very low detection of full length AR but robust staining for amino, containing variants (S3 Fig). These observations indicate that progression at castrate androgen levels may select against the full length AR but not AR variants.

### The transcriptional activity of full length androgen receptor is potentiated by AR variants

**Fig 3:**
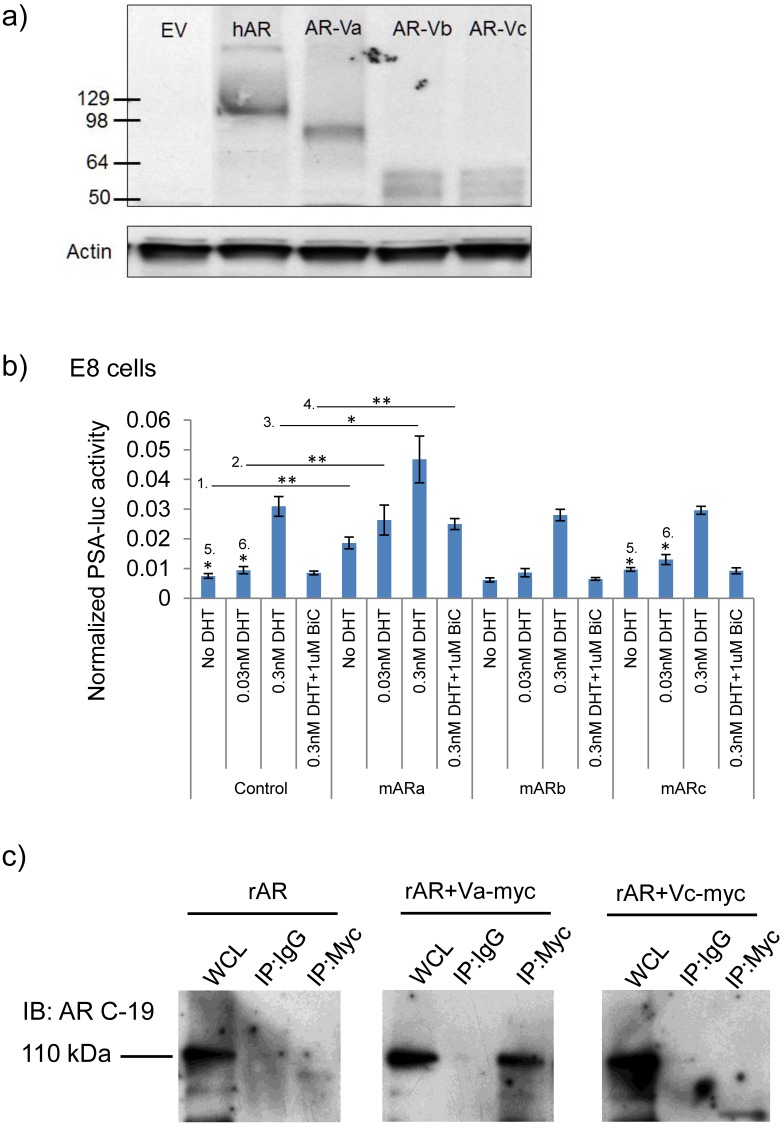
The transcriptional activity of full length androgen receptor is enhanced by AR variants. **a)** Western blotting using the AR (N-20) antibody confirms expression of cloned AR variants in COS-1 cells. **b)** Overexpression of AR variants in E8 cells treated with control, DHT (0.03, 0.3 nM) or DHT + Casodex (BiC, 1μM). (Control *vs*. mAR-Va = comparisons 1–4; Control *vs*. mAR-Vc = comparisons 5–6) (*, p<0.05, **, p<0.01). **c)** Co-immunoprecipitations from COS-1 cells transfected with AR-FL and either AR-Va-myc (middle) or AR-Vc-myc (right).

Our *in vivo* analysis suggests that the relative expression of AR isoforms can persist during CRPC and prompted us to examine the implications of a relative increase in expression of mAR-Vs in Pten deficient prostate cancer. To do this, we conducted a series of transcriptional assays using the PSA-luciferase reporter plasmid [28]. Firstly, each variant and the AR-FL were sub cloned into expression constructs followed by expression validation using western blotting and the AR (N-20) antibody (Fig 3a).

We found control E8 cells to be responsive to DHT in a dose dependent manner (0.03–0.3 nM) (Fig 3b) while the cE1 cells were not responsive to DHT unless exogenous rat AR-FL was introduced through transfection (S4 Fig). We next over-expressed mARVa, b, or c into E8 and cE1 cells and observed that the mAR-Va isoform significantly increased transcriptional reporter activity compared to control plasmid transfections (bar graph comparisons 1–4, *, p<0.05, **, p<0.01). Interestingly, mAR-Vc transfection augmented reporter levels only in the presence of androgens (comparisons 5–6; *, p<0.05) while mAR-Vb had a statistically insignificant effect (p>0.05) (Fig 3b).

To understand a potential mechanism by which mAR-Vs activate the function of AR-FL, we performed co-immunoprecipitations assaying for variant to AR-FL interaction. Plasmids encoding myc-tagged mAR-Va and mAR-Vc were generated for this assay based on the finding that these two ARVs could co-activate AR-FL in one of more of the cell lines tested. AR negative, COS-1 cells were transfected with rat AR alone (+ empty vector) or in combination with mAR-Va-myc (Va-myc) or mAR-Vc-myc (Vc-myc) expression constructs followed by immunoprecipitations for mAR-Va/Vc proteins using the myc epitope. Western blot analyses were then conducted using an antibody specific for the C-terminus of AR which only recognizes AR-FL. We found the 110 kDa AR-FL band to be present in the Va-myc immunoprecipitates indicating that mAR-Va could form a complex with AR-FL. However, since a significant AR-FL band was not detected in the Vc-myc immunoprecipitates, we inferred that stable complexing was less efficient or not formed between AR-FL and AR-Vc–an observation that is consistent with significantly reduced AR-Vb and AR-Vc mediated co-activation of the PSA reporter (Fig 3c). These data suggest that enhanced expression of mAR-Vs may enhance the stability of AR-FL thereby enhancing androgen dependent reporter assay output. These observations are also similar to those obtained with human AR-V5, 6, 7es [27].

**Fig 4:**
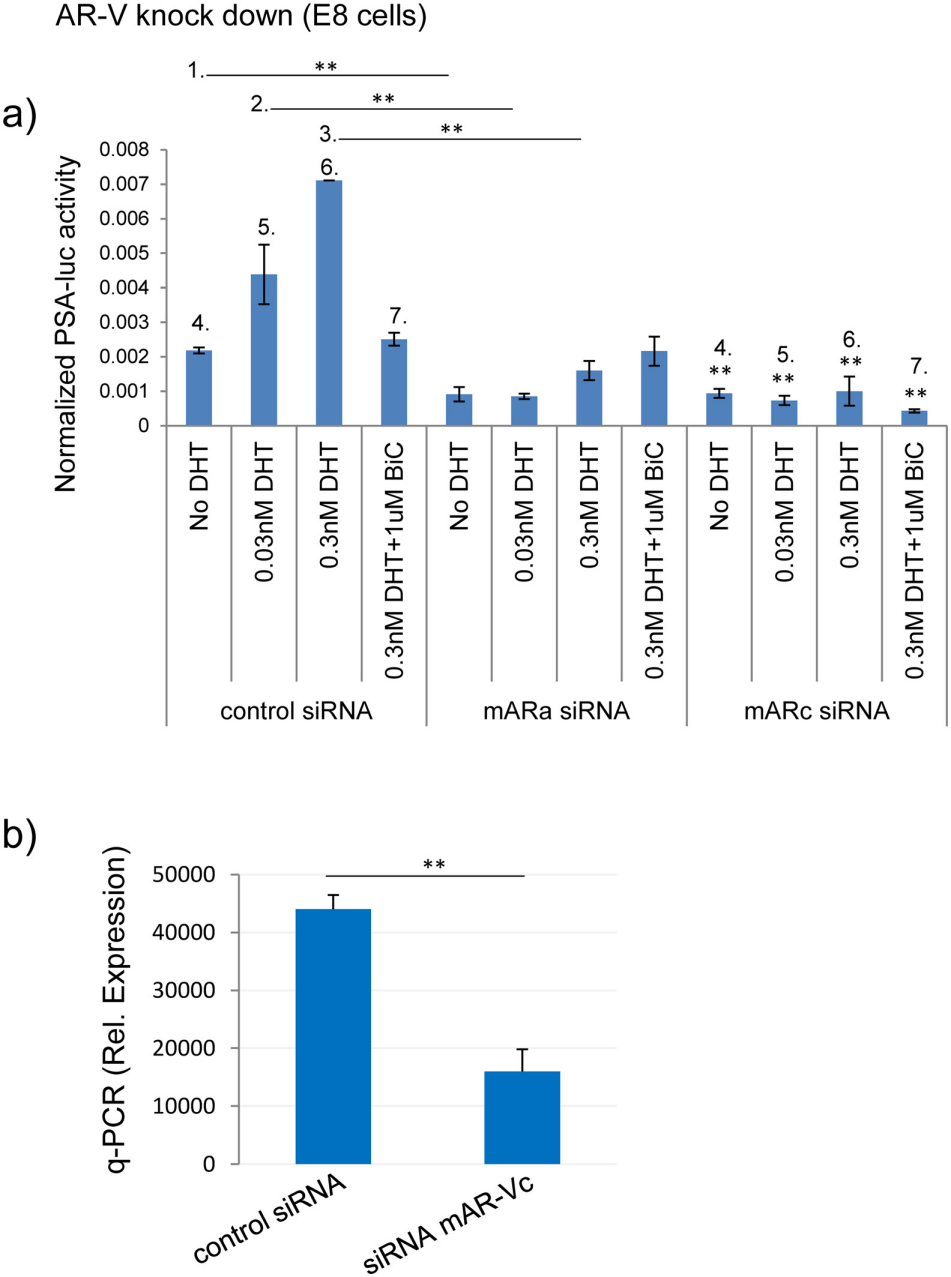
Knock down of androgen receptor variants regulates AR transcriptional activity in Pten deficient murine prostate cancer cell lines. **a)** E8 cells were transfected with siRNAs targeting either mAR-Va or c and evaluated for PSA-luc reporter activity 48 hours later (control siRNA *vs*. mAR-Va siRNA = comparisons 1–3; control siRNA *vs*. mAR-Vc siRNA = comparisons 4–7). b) Validation of siRNA knockdown of mAR-Vc as measured by q-PCR (*, p<0.05, **, p<0.01).

We then considered the effects of variant inhibition on PSA transcriptional reporter output. To do this we designed DICER substrate siRNAs (DsiRNAs) that would specifically target mAR-Va or c, but not full length AR. Through transfection of these siRNAs into E8 or cE1 cells along with the luciferase reporter plasmids, it was observed that knockdown of either mAR-Va and Vc reduced AR transcriptional activity in both cell lines (Fig 4a). Variant siRNAs were equally potent in E8 cells while in cE1 cells, knockdown of mAR-Vc yielded the most robust effect (data not shown). The efficiency of variant knock down is exemplified by q-PCR measurement of mAR-Vc levels in control and mAR-Vc-siRNA treated E8 cells (Fig 4b).

These results indicate that the mAR-Vs contribute to AR transcriptional activity in both E8 and cE1 cells and that their inhibition opposes AR activity. To further explore how the activity of AR signaling may be modified during CRPC, we measured expression levels of the AR target gene, *Fkbp5* [29], using two different *in vivo* manipulations. First, we castrated *C* ^*+*^ *;Pten* ^*L/L*^ mutants at 6 wks and analyzed mice at 30 wks and second we deleted epithelial AR in Pten deficient prostates (*C* ^*+*^ *;Pten* ^*L/L*^ *;Ar* ^*L*^ */Y*)—a model we have previously characterized (S5 Fig) [30]. Similar to our enzalutamide treated mutants (S3 Fig), we observed reduced/loss of expression of the distal AR targeting antibody (AR-C19) and maintained expression of the amino terminal targeting antibody (AR-N20). Compared to hormone intact (AR+) regions (S5b Fig, top left panel, AR+ arrows), FKBP5 expression was significantly diminished in castrate samples. As genetic validation and control for FKBP5 androgen dependent modulation, we assessed AR and FKBP5 expression in Pten-null;AR-null regions of *C* ^*+*^ *;Pten* ^*L/L*^ *;AR* ^*L*^ */Y* mutants observing complete loss of epithelial AR (AR- region) and near loss of FKBP5 protein expression (S5b Fig, top left, AR- arrows). Further, using CRPC lines derived from the Pten null mouse model [30], we validated androgen activation of *Fkbp5* upon addition of exogenous androgen (S5c Fig). These data indicate that during surgical or genetic induction of CRPC in Pten-null tumorgenesis, the AR-FKBP5 signaling axis is impaired.

## Discussion

Overexpression of androgen receptors (ARs) occurs in a significant number of late stage prostate cancers including those progressing to castration resistance. The identification of AR variants in human CRPC cell lines and clinical samples suggests that AR variants may facilitate continued AR oncogenic function despite the presence of AR targeted therapy [31]. This may be attributed to the fact that the majority of AR-Vs identified so far are predicted to encode proteins lacking the LBD but retaining the N-terminal transactivation domain thereby acting as the constitutively active transcription factors. This indicates that the AR LBD is dispensable for AR transcriptional activity and continued contribution towards cancer progression [27] [32] [33]. As a consequence, understanding the process by which AR variants arise and their contribution towards the emergence of CRPC has become an area of intense investigation.

Homologous systems of prostate cancer (hormone dependence, castration resistance and derived cell lines) as obtained using the Pten null model, provide an excellent platform to assess the disease initiation and progression [22]. Our study, for the first time, describes the natural occurrence of these AR NTD variants in Pten deficient mouse prostate cancer cells and the primary organ site. Importantly, we show that the identified AR-Vs are not static but altered in expression during CRPC progression. The three mouse AR variants identified in this study are composed of novel molecular structures. mAR-Va, which retains the contiguous exons 1–4 followed by read-through into intron 4 and is roughly analogous to the reported mAR-V4 [15] and AR^V567es^ [27] with the exception of the unique amino acid sequence encoded by the 3’-end. mAR-Vb and mAR-Vc are composed of two different regions located within intron 1 spliced after exon 1, leading to the deduced AR-Vs protein sequence containing only the NTD. The so called AR-V8 identified in 22rv1 human prostate cancer cells appears as the closest human counterpart containing the AR-NTD followed by a longer 33-aa tail encoded by an exon 3’ located within intron 3 [19]. The similarity between identified mouse variants and those previously identified in human cell lines, suggests that the cellular stress of hormone ablation therapy may act in common manner during selection for variants AR variant populations

Recent clinical studies have demonstrated hAR-V7 to be elevated in CRPC, metastasis, association with biochemical recurrence and poor clinical outcome [11] [17] [18] [34]. One of the most significant findings from our study is the ability of castration or AR targeted therapy to select for mouse AR isoforms. These data not only demonstrate an additional mechanism by which *Pten* deficient prostate cancer cells can progress to CRPC but also has strong implications for therapies intended to directly target the ligand binding domain of the androgen receptor. Interestingly, while we observed enhanced relative expression of mARVs in derived cell lines in culture, expression of AR-FL was maintained which is different from what was observed in the mouse primary tumors. This may be attributed to the monoclonal nature of cell derivation *versus* the highly heterogeneous nature of primary samples. It may also be associated with general maintenance of cell lines in hormone intact conditions *versus* the long castrate conditions used to maintain primary tumors. The striking observation that AR-FL decreased during progression at castrate androgen levels or in the presence of chronic enzalutamide exposure, provides compelling support that AR-FL is selected against and that ARVs may facilitate continued AR function. Previous reports have shown that *Pten* null mutants are poorly responsive to both castration and enzalutamide therapy [23] [35] [24]. Our data demonstrated the relative increase of AR-NTD variants, both *in vitro* and *in vivo*, which is consistent with a lack of response to AR targeted therapy in this model.

We observed the capacity for mAR-Va, but reduced for mAR-Vb and mAR-Vc, to enhance the activity of AR transcriptional reporter assays. This may be attributed to fact that mAR-Vb and Vc lack a DNA binding motif. Our data, however, cannot rule out the potential for variants such as mAR-Vb and Vc to have oncogenic function through alternative mechanisms. Interesting, a novel AR variant of similar structure was identified in several human prostate cancer cell lines [19]. The so called AR8 also lacks a DNA binding domain and, thus, like the mAR-Vb and mAR-Vc identified here, is unlikely to function as a transcriptional factor on its own. Rather, AR8 was shown to associate with Src and the EGF receptor thereby promoting enhanced phosphorylation of the AR [19]. It will be interesting for future studies to address whether mAR-Vb and Vc are capable of similar plasma membrane associations and a potential contribution to CRPC.

Our AR-V knock down studies, which resulted in reduced AR transcriptional activity, reveal potential therapeutic applications of variant targeting during progression to CRPC. The presence of truncated variants in Pten deficient mouse is also consistent with the clinical observation maintained AR expression despite the presence of potent AR targeted therapies such enzalutamide. However, since mAR-Va, b, and c showed differential responses in expression changes to castration or casodex in our studies, the exact contribution(s) of individual variants, and their potential cooperativity towards CRPC remains to be elucidated and likely depend on cellular context [8]. Future progression studies may consider mAR-V cellular localizations, interactions with other oncogenic pathways and impact on *in vivo* progression.

Findings presented here provide evidence for the presence of novel variants detectable in the primary organ system of genetically engineered Pten null mice highlighted by the enhanced variant expression during progression at castrate androgen levels. Future therapeutic studies may consider whether early targeting of these identified AR isoforms may prevent or delay progression to CRPC. That mAR-Va and Vc inhibition resulted in reduced AR transcriptional function provides the rationale for testing of pharmacological targeting AR variants. Given our results, it is tempting to speculate that introduction of AR-FL back to certain Pten deficient CRPC cells containing AR Vs may restore such cells to androgen deprivation therapy.

## Methods

### Cell culture and mouse prostate samples

E4, E8, cE1 cE2 [25] [26] and CaP8, P8 [36] cell line were established from the conditional *Pten*-null mouse model. LNCaP and PC-3 cells were purchased from ATCC. C4-2B and COS-1 cells were a gift from Dr. Baruch Frenkel (University of Southern California, LA, CA) and Dr. Allen Epstein (University of Southern California, LA, CA), respectively. The derivatives of parental cell lines were generated through culture 10% CSS (Charcoal-stripped Serum, Cellgro) for up to 18 days. E8 and cE2 cells were also treated in normal maintaining media with the addition of 10 μM of anti-androgen, bicalutamide (Sigma-Aldrich) for 6 days. Prostatic tissues were collected from mice bearing ADCa or CRPC tumors as well as wild-type ones at different age groups. Dissected prostates were processed by rapid freezing in liquid nitrogen and stored in -80°C for further analysis.

### Cloning and constructs

Total cellular RNAs were isolated from E8 and cE1 by RNAqueous-4PCR Kit (Life technologies) used as the source. The detection of mouse AR splice variants from them was achieved by GeneRacer Kit with SuperScript III RT and TOPO TA Cloning Kit for Sequencing (life technologies) as per manufacture’s protocols. Briefly, extracted RNAs were first reverse transcribed using the GeneRacer oligo (dT) primer and SuperScript III RT module from the kit. RACE-ready cDNAs were subjected to the 3’ rapid amplification of cDNA end PCR (3’ RACE-PCR) using a forward gene-specific primer (GSP) and reverse GeneRacer 3’ primer from the GeneRacer Module. Another round of nested PCR was conducted for further amplification using a forward GSP nested primer and reverse GeneRacer 3’ Nested primer provided in the kit. SuperTaq Plus Polymerase (Life technologies) was used for both 3’RACE and nested PCR. Forward GSP and nested primers anchored within exon1 (S1 Table) for predicted variants (S2 Table) using variant specific RACE primers (S3 Table). 3’ RACE and nested PCR products were then examined by standard TA sub cloning and sequencing using the TOPO TA Cloning Module. ApE plasmid Editor was used in this study to analyze sequencing results, predict protein sequences and generate text maps for mARVs (S3 Table).

Plasmid constructs used in this study included: Rat AR full-length (Dr. Robert Matusik, Vanderbilt University, Nashville, TN), human AR full-length (Dr. Jeremy Jones, City of Hope, Duarte, CA), and PSA-luciferase reporter and PCDNA3.1-myc (Dr. Parkash Gill, University of Southern California, LA, CA). pCR4-TOPO was purchased in the TOPO TA Cloning Kit for Sequencing (life technologies).

CDS (coding sequences) for mARVa, Vb and Vc were amplified by PCR from cDNAs of E8 and cE1 using the primer sets enclosing the entire open reading frame (ORF), and sub cloned into pcDNA3.1-myc-HisC. To generate the ARVa-myc and ARVc-myc constructs, reverse primers were re-designed to be anchored upstream of their own stop codons and in-framed with the ORF of expression vector. Hifi AccuStart Taq DNA Polymerase (Quanta Biosciences) and HotStar Taq DNA Polymerase (Qiagen) were applied in PCR cloning. Sequences of primer used to clone Va, Vb, Vc, Va-myc and Vc-myc were provided in S4 Table.

### PCR and Quantitative real-time PCR

Total cellular RNAs were extracted from all parental and other derivative cell lines and then reverse transcribed using oligo (dT) primer and SuperScript III First-Strand Synthesis (Life technologies). RNA samples used for various assays were isolated from cells with different batches. Conventional PCR was performed on cDNAs using the same forward primer anchored within exon1 paired with reverse primer specific for mAR-Va, b, c and AR-FL, respectively (S5 Table). Quantitative real-time PCR and data analysis have been described previously. The real-time primer sets designed specifically and exclusively to amplify each ARV and AR-FL were listed in S6 Table.

### Transfection and luciferase reporter assay

1–2 days prior to transfection, cells were placed in media containing charcoal-stripped serum (CSS). For all transfections, pools of cells were transfected using Lipofectamine Plus (Invitrogen) with empty vector control plasmid, full-length AR plasmid, or AR splice variant plasmids along with the androgen-responsive constructs MMTV-firefly luciferase or PSA-firefly luciferase [28] and the androgen-insensitive renilla luciferase control pRL-SV40 (Promega). Empty vector control plasmid was used to equilibrate total DNA in all transfections. The following day, the cells were plated in quadruplicate with drugs in 96 well plates. 24hrs later luciferase activities were quantified (Dual luciferase assay kit, Promega) on a plate reader (Tecan) and the firefly signal normalized to the renilla signal to control for cell number.

### Co-Immunoprecipitation assay

COS cells were transfected with mARVa-myc or mARVc-myc together with Rat AR-FL using Lipofectamine 2000 (Life technologies) as per the manufacture’s protocol. About 48 hrs. post-transfection cells were lysed with 1% CHAPS lysis buffer (10 mM Tris-HCl, pH 7.4, 150 mM NaCl, 0.5 mM CaCl2, 0.5 mM MgCl2 supplemented with protease inhibitor cocktail (Pierce). Then 500 μl cleared cell lysate were immunoprecipitated by incubation with 5 μg anti-myc mAb (Roche) and Protein G PLUS-Agarose (EMD Millipore) overnight at 4°C. The isotype matched control mAbs were included in all immunoprecipitation experiments as negative control. Immunoprecipitates were washed twice in 0.1% lysis buffer followed by elution separation on 4–20% Criterion Tris-HCl Gel (Bio-Rad) under reduced conditions.

### Western blot analysis

Whole-cell lysates from cell lines and primary tissues were prepared as described previously [37]. Protein samples were loaded on 10% polyacrylamide gels (Thermo Scientific) and subjected to immunoblot analysis with anti-AR (N20; Santa Cruz). After detection of signals membranes were stripped and re-probed against actin (Santa Cruz) to confirm equivalent loading and transfer of protein. The elutions from the co-immunoprecipitation were resolved by SDS-PAGE and analyzed by western blot. The antibodies used in this study included AR (N-20, Santa Cruz), AR (C19, Santa Cruz), Myc and Actin (Santa Cruz). The molecular weight and expression levels of protein samples were assessed by Gel Doc XR+ system (Bio-Rad).

### Use of mouse lines

All mice were maintained in a temperature and humidity controlled, pathogen free housing units, with light-dark cycles (10 hours light, 14 hour dark) allowing food and water distribution *ad libitum*. Breeding and experimental procedures were performed according to mouse protocols approved by the Institutional Animal Use and Care Committee (IACUC) at the University of Southern California and Icahn School of Medicine, Mount Sinai. The current study is specifically approved under the protocol: “The Function of the PTEN tumor suppressor gene in tumorgenesis and animal development” with Lab Animal Protocol Reference #LA13-00060. Conditional Pten-null mutant mice (*C* ^*+*^ *;Pten* ^*L/L*^) and Pten-null;Ar-null mutant mice were carried on a pure C67/BL6 background Only male mice were used for experimental procedures.

### Animal surgical procedures

Surgical castration recovery surgery was carried out in Pten mutant mice while under full anesthetic using Ketamine (50 mg/ml) and Xylazine (20 mg/ml) according to an approved animal protocol (Reference #LA13-00060).

## Supporting Information

S1 Fig Schematic and transcript sequence of AR-Va, AR-Vb and AR-Vc. Sequences were obtained through 3’ RACE PCR on cDNA generated from murine prostate cancer cell lines (E8, cE1). RACE PCR was followed by nested PCR to identify new AR variants.
(PDF)

S2 Fig Relative expression of AR variants in Pten null, murine prostate cancer cell lines. (PDF)

S3 Fig Low expression of full length androgen receptor in enzalutamide treated mutants. Pten mutants (*C* ^*+*^ *;Pten* ^*L/L*^, n = 5) were treated for >20 wks with enzalutamide and then assessed for AR expression using antibodies against the animo terminus (AR, N-20) or carboxyl terminus (AR, C-19). Significant nuclear expression is observed with the N-20 antibody but only low expression with the C-19.
(PDF)

S4 Fig AR-variant dependent activation of the PSA transcriptional reporter in the cE1 prostate cancer cell line. Presence of AR-FL is required for transactivation of the PSA-luc reporter (*, p<0.05, **, p<0.01) (S4a Fig). **b**Variant dependent PSA-luc transactivation (mARa, comparisons 1–4; mAR-Vc, comparisons 5–6) (*, p<0.05, **, p<0.01) (S4b Fig).
(PDF)

S5 Fig FKBP5 expression in Pten mutant CRPC prostate tissue and cell lines. a) Pten null mutants were castrated (*C* ^*+*^ *;Pten* ^*L/L*^, 6 wks) and assessed at 30 wks for AR (AR-Nt, AR-Ct) and FRKBP5 expression (S5a Fig). Hormone intact Pten-null;Ar-null (*C* ^*+*^ *;Pten* ^*L/L*^ *;Ar* ^*L*^ */Y*) mutants assessed at 30 wks for AR (AR-Nt, AR-Ct) and FRKBP5 expression (S5b Fig). CRPC Pten-null cell lines derived from the Pten-null mouse model assessed for *Fkbp5* gene expression in hormone intact (FBS), castrate (CSS) or with exogenous androgen (1 nM R1881) over 20 hours treatment (S5c Fig).
(PDF)

S1 Table RACE PCR forward anchored PCR primers. (PDF)

S2 Table Predicted size of AR variants. (PDF)

S3 Table RACE PCR primers for individual variants. (PDF)

S4 Table Q-PCR primers for individual variants. (PDF)

S5 Table PCR primers for cloning of mAR-Va, b, c. (PDF)

S6 Table PCR primers for cloning of mAR-Va-myc and mAR-Vc-myc. (PDF)
