# Correction: Identification of Androgen Receptor Splice Variants in the Pten Deficient Murine Prostate Cancer Model

**DOI:** 10.1371/journal.pone.0152243

**Published:** 2016-03-21

**Authors:** Mengmeng Liang, Helty Adisetiyo, Xiuqing Li, Ren Liu, Parkash Gill, Pradip Roy-Burman, Jeremy O. Jones, David J. Mulholland

The third author’s name is spelled incorrectly. The correct name is: Xiuqing Li. The correct citation is: Liang M, Adisetiyo H, Li X, Liu R, Gill P, Roy-Burman P, et al. (2015) Identification of Androgen Receptor Splice Variants in the Pten Deficient Murine Prostate Cancer Model. PLoS ONE 10(7): e0131232. doi:10.1371/journal.pone.0131232.
