# Supplementary figures and images for: Identification of Androgen Receptor Splice Variants in the Pten Deficient Murine Prostate Cancer Model
Source: PLoS One. 2015 Jul 21;10(7):e0131232. doi: 10.1371/journal.pone.0131232 (PMC4510390; doi:10.1371/journal.pone.0131232)

## a) Variant AR Va

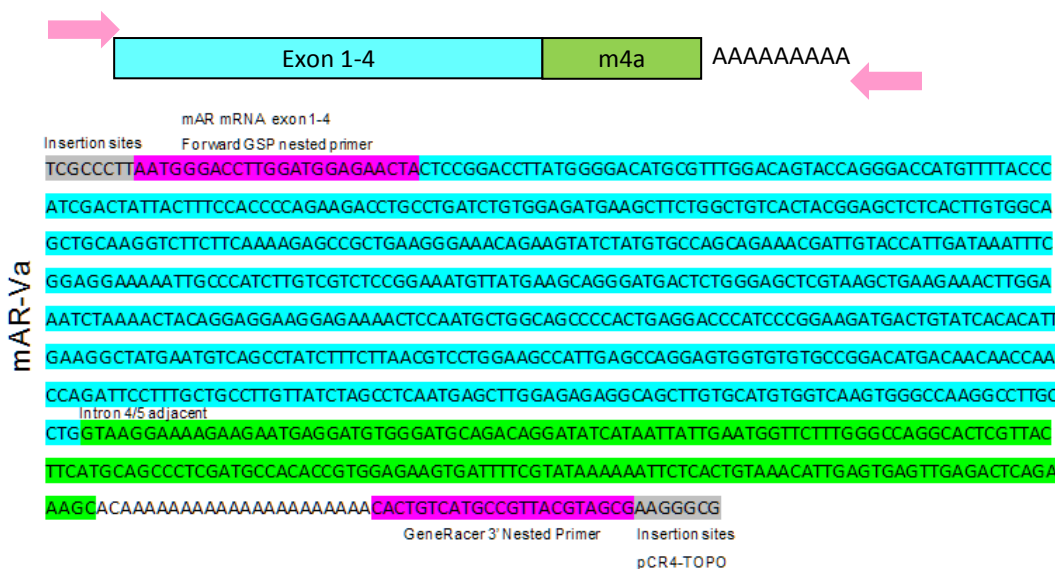

## b) Variant AR Vb

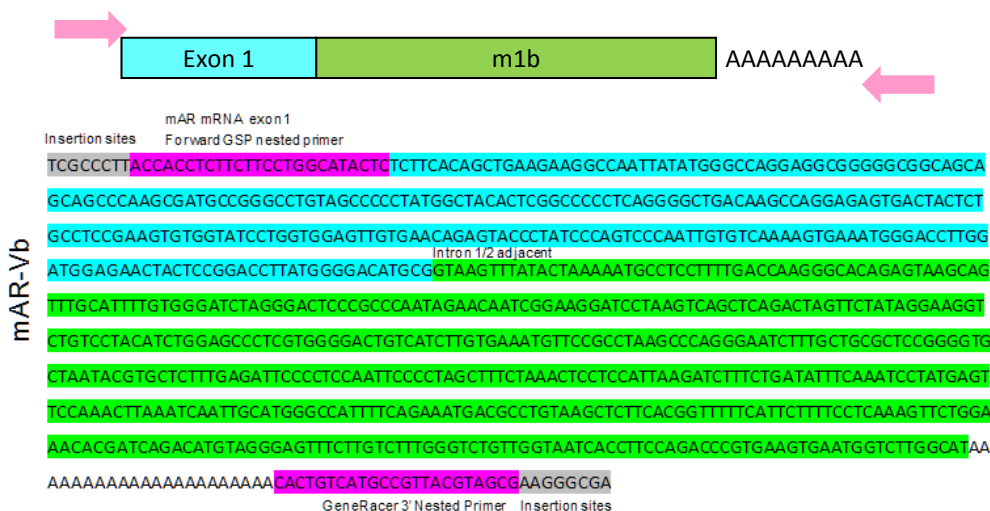

## c) Variant AR Vc

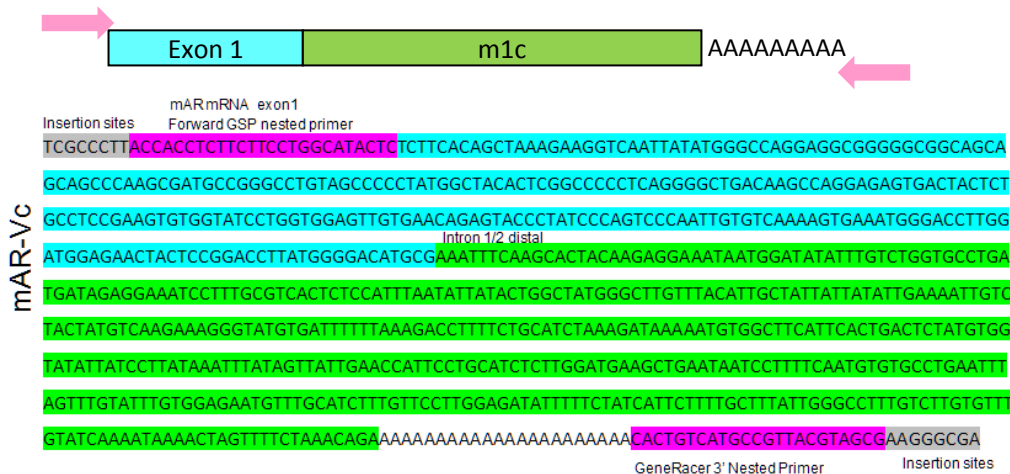

Supplement: S1 Fig — Sequences were obtained through 3’ RACE PCR on cDNA generated from murine prostate cancer cell lines (E8, cE1). RACE PCR was followed by nested PCR to identify new AR variants. (PDF) [file pone.0131232.s001.pdf]

Fig S2

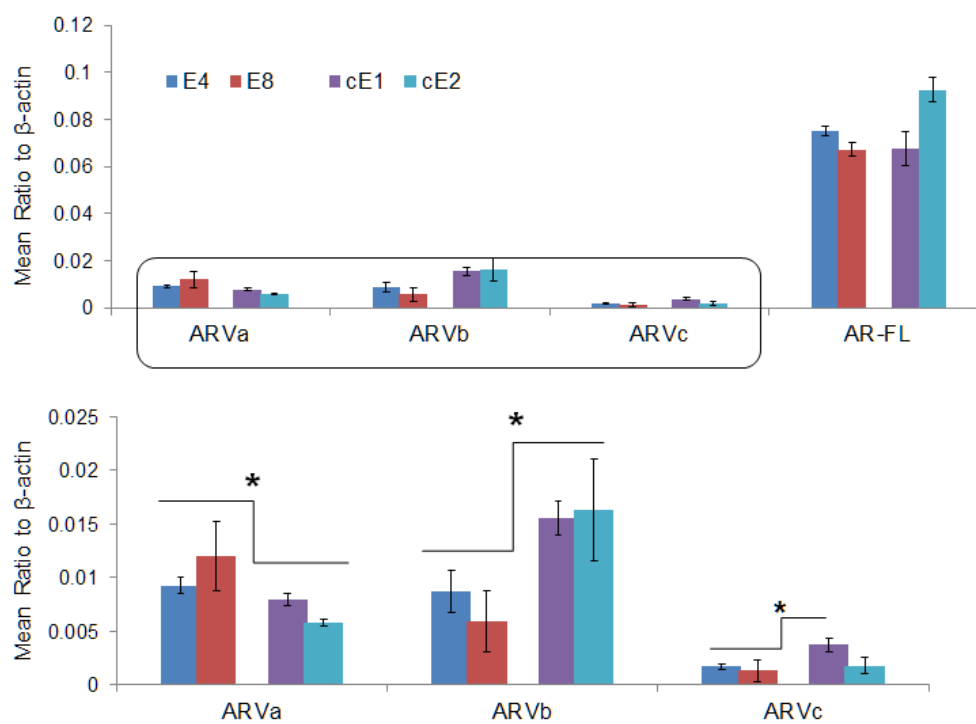

Supplement: S2 Fig — (PDF) [file pone.0131232.s002.pdf]

Fig S3

$C^+;Pten^{L/L}$  (Enzalutamide, 10 mg/kg, 10 wks)

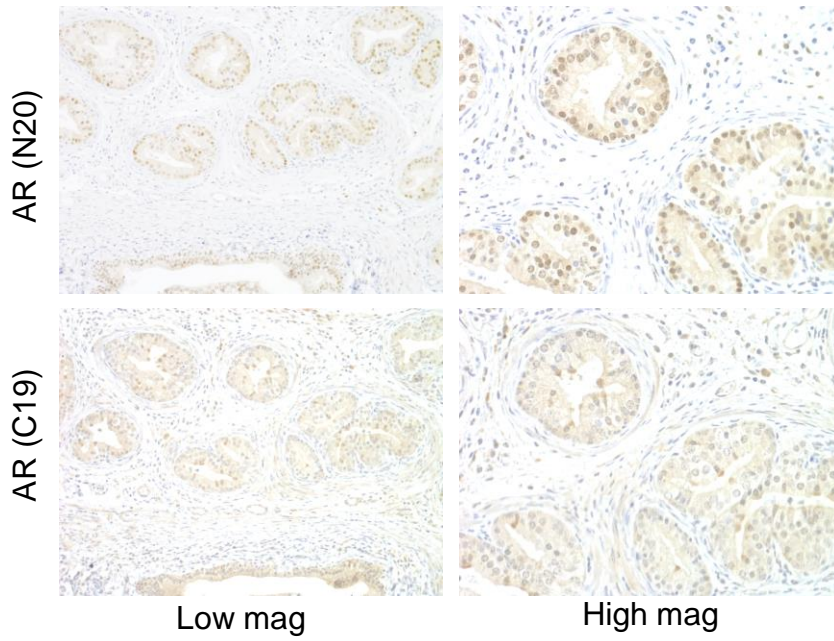

Supplement: S3 Fig — Pten mutants (C + ;Pten L/L, n = 5) were treated for >20 wks with enzalutamide and then assessed for AR expression using antibodies against the animo terminus (AR, N-20) or carboxyl terminus (AR, C-19). Significant nuclear expression is observed with the N-20 antibody but only low expression with the C-19. (PDF) [file pone.0131232.s003.pdf]

a) cE1 cell line

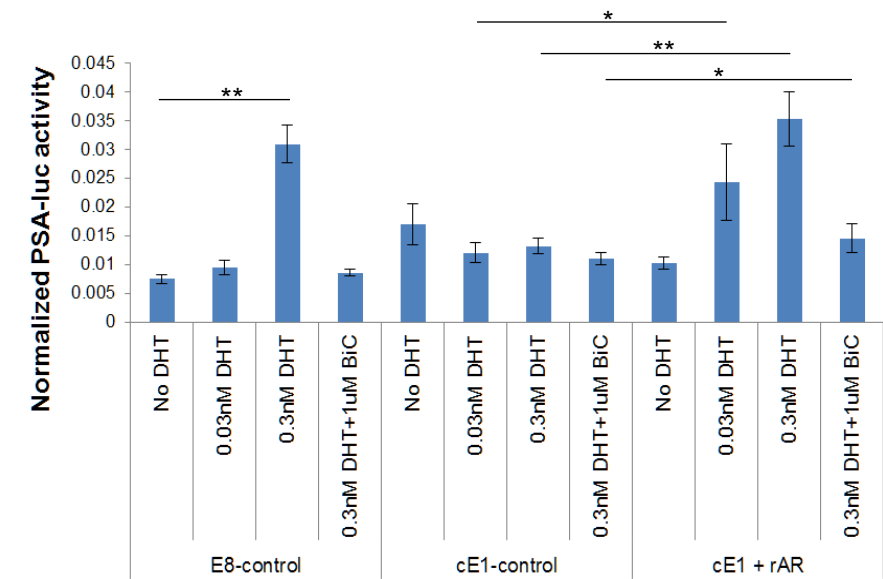

b) cE1 cell line

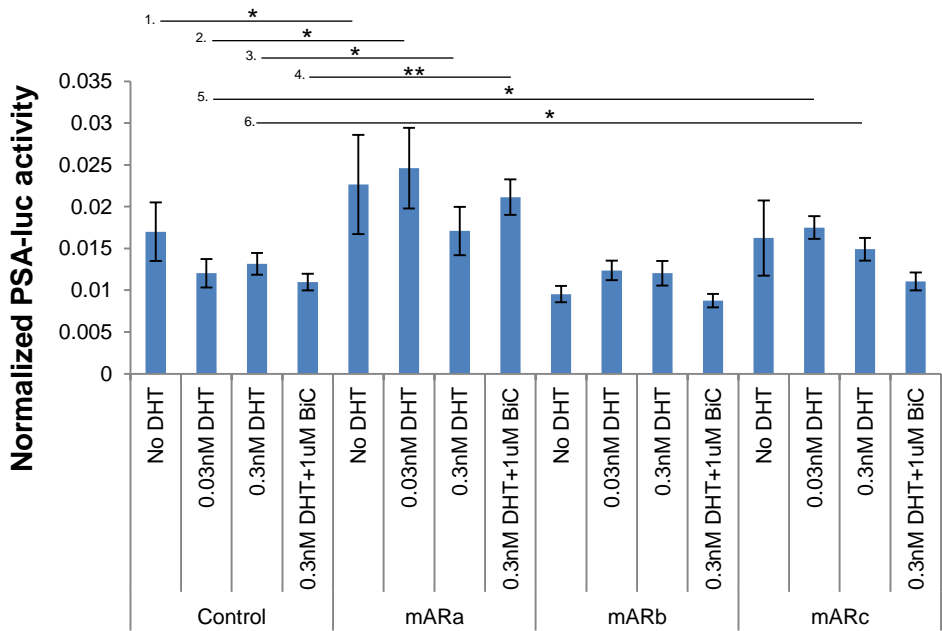

Supplement: S4 Fig — Presence of AR-FL is required for transactivation of the PSA-luc reporter (*, p<0.05, **, p<0.01) (S4a Fig). bVariant dependent PSA-luc transactivation (mARa, comparisons 1–4; mAR-Vc, comparisons 5–6) (*, p<0.05, **, p<0.01) (S4b Fig). (PDF) [file pone.0131232.s004.pdf]

a)  $C^+;Pten^{L/L}$  (castrate), >30 wks

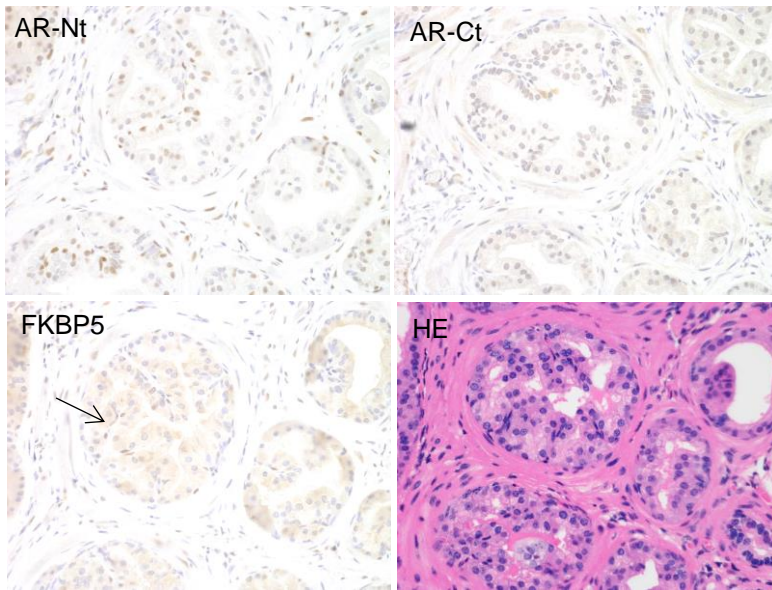

b)  $C^+;Pten^{L/L};Ar^L/Y$  (Intact), >30 wks

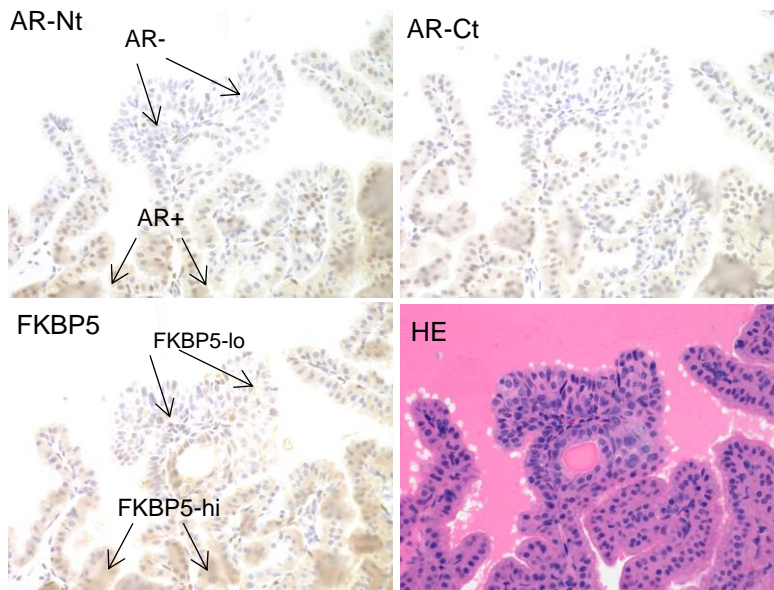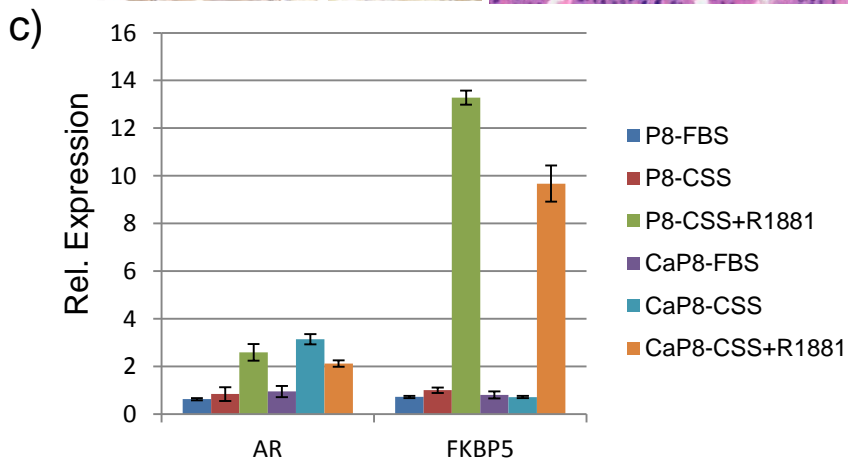

Supplement: S5 Fig — a) Pten null mutants were castrated (C + ;Pten L/L, 6 wks) and assessed at 30 wks for AR (AR-Nt, AR-Ct) and FRKBP5 expression (S5a Fig). Hormone intact Pten-null;Ar-null (C + ;Pten L/L ;Ar L /Y) mutants assessed at 30 wks for AR (AR-Nt, AR-Ct) and FRKBP5 expression (S5b Fig). CRPC Pten-null cell lines derived from the Pten-null mouse model assessed for Fkbp5 gene expression in hormone intact (FBS), castrate (CSS) or with exogenous androgen (1 nM R1881) over 20 hours treatment (S5c Fig). (PDF) [file pone.0131232.s005.pdf]
