# Supplementary material for: Identification of Androgen Receptor Splice Variants in the Pten Deficient Murine Prostate Cancer Model
Source: PLoS One. 2015 Jul 21;10(7):e0131232. doi: 10.1371/journal.pone.0131232 (PMC4510390; doi:10.1371/journal.pone.0131232)
Supplement: S1 Table — (PDF) [file pone.0131232.s006.pdf]

Table 1. RACE PCR forward anchored PCR primers.

	Exon Location
Forward GSP primer	TCTGTCTCTGTATAAATCTGGAGCA
Forward GSP Nested primer	ACCACCTCTTCTTCCTGGCATACTC
