# Supplementary material for: Identification of Androgen Receptor Splice Variants in the Pten Deficient Murine Prostate Cancer Model
Source: PLoS One. 2015 Jul 21;10(7):e0131232. doi: 10.1371/journal.pone.0131232 (PMC4510390; doi:10.1371/journal.pone.0131232)
Supplement: S2 Table — (PDF) [file pone.0131232.s007.pdf]

Table 2. Predicted size of AR variants.

	mRNA (bp)	amino acid	MW (KD)	Cell Origin
AR-Va	2320	746	~80	E8
AR-Vb	2081	518	~54	cE1
AR-Vc	2029	526	~56	cE1
