# Supplementary material for: Identification of Androgen Receptor Splice Variants in the Pten Deficient Murine Prostate Cancer Model
Source: PLoS One. 2015 Jul 21;10(7):e0131232. doi: 10.1371/journal.pone.0131232 (PMC4510390; doi:10.1371/journal.pone.0131232)
Supplement: S3 Table — (PDF) [file pone.0131232.s008.pdf]

Table 3. RACE PCR primers for individual variants.

	Forward	Reverse	Expected Product
AR-Va	AGCCACCACCTCTTCTT CCT	GCATCCCACATCCTCATTCT	879 bp
AR-Vb		AAGATGACAGTCCCCACGAG	494 bp
AR-Vc		AATGGAGAGTGACGCAAAGG	401 bp
AR-FL		TACTGAATGACCGCCATCTG	874 bp
