# Supplementary material for: Identification of Androgen Receptor Splice Variants in the Pten Deficient Murine Prostate Cancer Model
Source: PLoS One. 2015 Jul 21;10(7):e0131232. doi: 10.1371/journal.pone.0131232 (PMC4510390; doi:10.1371/journal.pone.0131232)
Supplement: S4 Table — (PDF) [file pone.0131232.s009.pdf]

Table 4. q-PCR primers for individual variants.

	Forward	Reverse	Expected Product
AR-Va	TCCTTTGCTGCCTTGTTATC	GCATCCCACATCCTCATTCT	117 bp
AR-Vb	GGGACCTTGGATGGAGAACT	GGCGGAACATTTCACAAGAT	238 bp
AR-Vc	AGTGAAATGGGACCTTGGAT	AAATGGAGAGTGACGCAAAG	139 bp
AR-FL	TCCAGGATGCTCTACTTTGC	TTTTGATTTTTCAGCCCATC	200 bp
