# Supplementary material for: Identification of Androgen Receptor Splice Variants in the Pten Deficient Murine Prostate Cancer Model
Source: PLoS One. 2015 Jul 21;10(7):e0131232. doi: 10.1371/journal.pone.0131232 (PMC4510390; doi:10.1371/journal.pone.0131232)
Supplement: S5 Table — (PDF) [file pone.0131232.s010.pdf]

Table 5. PCR primers for cloning of mAR-Va, b, c

	Forward	Reverse
ARVa	ACGTGAATTCGAAGCTACAGACAAGCTCAA	ACGTCTCGAGCTGAGTCTCAACTCACTCAA
ARVb		ACGTCTCGAGTTGGTCAAAAGGAGGCATTT
ARVc		ACGTCTCGAGATTTCCTCTATCATCAGGCA
