# Supplementary material for: Identification of Androgen Receptor Splice Variants in the Pten Deficient Murine Prostate Cancer Model
Source: PLoS One. 2015 Jul 21;10(7):e0131232. doi: 10.1371/journal.pone.0131232 (PMC4510390; doi:10.1371/journal.pone.0131232)
Supplement: S6 Table — (PDF) [file pone.0131232.s011.pdf]

Table 6. PCR primers for cloning of mAR-Va-myc and mAR-Vc-myc.

	**Forward**	**Reverse**
mAR-Va-myc	ACGTGAATTCGAAGCTACAGACAAGCTCAA	ACGTCTCGAGCTACGAAAATCACTTCTCCA
mAR-Vc-myc		ACGTCTCGAGCTTTCCTCTTGTAGTGCTTGAAATTTCGCATGTCCCCATAAGGT
